# Adsorption–Desorption Behavior of Hydrogen Sulfide Capture on a Modified Activated Carbon Surface

**DOI:** 10.3390/ma16010462

**Published:** 2023-01-03

**Authors:** Nurul Noramelya Zulkefli, Adam Mohd Izhan Noor Azam, Mohd Shahbudin Masdar, Wan Nor Roslam Wan Isahak

**Affiliations:** 1 Department of Chemical & Process Engineering, Faculty of Engineering & Built Environment, Universiti Kebangsaan Malaysia, Bangi 43600, Selangor, Malaysia; 2 Fuel Cell Institute, Universiti Kebangsaan Malaysia, Bangi 43600, Selangor, Malaysia; 3 Research Center for Sustainable Process Technology (CESPRO), Faculty of Engineering & Built Environment, Universiti Kebangsaan Malaysia, Bangi 43600, Selangor, Malaysia

**Keywords:** adsorbents, hydrogen sulphide, adsorption–desorption, behavioral model

## Abstract

Metal-based adsorbents with varying active phase loadings were synthesized to capture hydrogen sulfide (H_2_S) from a biogas mimic system. The adsorption–desorption cycles were implemented to ascertain the H_2_S captured. All prepared adsorbents were evaluated by nitrogen adsorption, Brunauer–Emmett–Teller surface area analysis, scanning electron microscopy–energy-dispersive X-ray spectroscopy, and Fourier transform infrared spectroscopy. From the results, modified adsorbents, dual chemical mixture (DCM) and a core–shell (CS) had the highest H_2_S adsorption performance with a range of 0.92–1.80 mg H_2_S/g. After several cycles of heat/N_2_ regeneration, the total H_2_S adsorption capacity of the DCM adsorbent decreased by 62.1%, whereas the CS adsorbent decreased by only 25%. Meanwhile, the proposed behavioral model for H_2_S adsorption–desorption was validated effectively using various analyses throughout the three cycles of adsorption–desorption samples. Moreover, as in this case, the ZnAc_2_/ZnO/CAC_OS adsorbents show outstanding performances with 30 cycles of adsorption–desorption compared to only 12 cycles of ZnAc_2_/ZnO/CAC_DCM. Thus, this research paper will provide fresh insights into adsorption–desorption behavior through the best adsorbents’ development and the adsorbents’ capability at the highest number of adsorption–desorption cycles.

## 1. Introduction

Fermentation of animal or agricultural waste in the absence of oxygen results in the production of biogas, a form of renewable energy. Heat energy derived from biogas can be directly converted into other forms of energy, and the byproduct can be utilized as fertilizer for agriculture. The primary components of biogas are 50%–70% CH_4_ and 30%–50% CO_2_, and the minor components consist of NH_3_ (80–100 ppm), H_2_S (500–1000 ppm), and a trace of hydrocarbon (100 ppm) [1]. The capability of biogas to directly generate electricity and heat energy has drawn significant attention for use in engineering applications. However, one of the challenges of biogas purification is that it results in the release of hydrogen sulfide (H_2_S), which might have a negative effect on the production, machinery and catalyst and result in health issues [2,3,4]. Hence, H_2_S removal requires biogas purification to improve its properties and make biogas comparable to natural gas as a fuel for automobiles.

H_2_S removal could be carried out in many ways using gas purification technology depending on how H_2_S is made up in the gas stream. Watanabe [5] looked closely at the liquid absorption, slurry reactors, and fixed-bed columns that are typically used to remove sulfur from natural gas. Alkylamine absorbents for sour gases [6] are often used in large facilities [7], but they are hard on the economy because of their high operating costs [8], problems with selectivity, and the way the liquid sorbent breaks down over time. Slurry reactors [9] allow good material exchange between solids and fluids at low operating temperatures. They may be a good alternative to fixed-bed reactors for sorbent recovery.

The latter has become well-known because of recent technological advances that have made them a true cornerstone in H_2_S capture, especially in the purification of biogas, a promising and renewable energy source made from the anaerobic digestion of organic wastes. Hence, desulfurization is often carried out through a process called adsorption, which is not as complicated as other methods and is thought to have a high removal efficiency [10]. The adsorption process can be divided into two distinct mechanisms, namely, physisorption and chemisorption, by which solid sorbents used in fixed-bed reactors for H_2_S capture may operate [11]. Chemisorption is based on the chemical bonds between the adsorbate and substrate, whereas physisorption involves the formation of weak bonds between H_2_S and the substrate. 

Various types of adsorbents have been investigated for H_2_S and CO_2_ adsorption to improve adsorption capability. These adsorbents include metal–organic frameworks, which have demonstrated high adsorption capacity and selectivity toward H_2_S removal [12,13,14,15], as well as CO_2_ [16] and zeolites, which are commonly applied in CO_2_ removal owing to their high selectivity, ability to regenerate, and thermal resistance; H_2_S separation is performed through chemisorption at very low partial pressures [17,18,19,20] or physisorption from low to moderate partial pressures [21,22,23,24,25]. 

Due to its high specific surface area and porous structure, activated carbon (AC) is commonly employed as an adsorbent for H_2_S. According to a number of studies [26,27,28], the surface properties of carbon (such as pH, functional groups, pore size distribution, and specific surface area) are considerably correlated with H_2_S removal. Generally, modified AC has gained much attention to enhance the capability of AC in captured H_2_S. Modified AC with metal and alkaline adsorbents has a considerable influence toward H_2_S adsorption capabilities compared with commercial AC [29,30,31,32]. Compared with unimpregnated adsorbents (commercial AC), the advantages of impregnated ACs for H_2_S removal are their high efficiency and rapid kinetics of reaction [32]. The impregnation technique is essential for ensuring the uniform distribution of chemicals on the surface of AC, preserving maximum access to the pore structure to provide the target contaminants (H_2_S) with maximum contact efficiency to the impregnated AC [33]. 

Chiang et al. [34] tested the performance on H_2_S adsorption using the impregnated AC technique with NaOH, Na_2_CO_3_, KOH, and K_2_CO_3_. The adsorbents’ performances were ranked as follows for H_2_S adsorption: NaOH > Na_2_CO_3_ > KOH > K_2_CO_3_. Yan et al. [33,35] tested alkaline AC under 1% H_2_S and 80% relative humidity (RH). After pre-humidification (RH = 100%), alkaline AC could hold more H_2_S than before. 

AC impregnated with iron (III) chloride is also capable of removing H_2_S selectively [36]. Transition metal oxides (e.g., ZnO, CuO, Mn_2_O_3_, Fe_2_O_3_, CeO_2_) and mixed transition metal oxides (e.g., zinc ferrite [ZnFe_2_O_4_], Zn-Ti-O, CuO-CeO_2_) were used as adsorbents for downstream hot gas desulfurization [37]. During the sulfidation stage, these metal oxides are typically transformed to metal sulfides and then consecutively regenerated by oxidation of metal sulfides to metal oxides. The use of ZnO was studied by Basyooni et al. [38] who showed its potential to demonstrate higher sensitivity towards CO_2_ and H_2_S gas in gas sensing. In another study, Basyooni et al. concluded that ZnO might be affected in positive ways in order to enhance the adsorption process when introduced in a similar to the impregnation technique [39]. However, Zulkefli et al. [29,30,31,32] confirmed that only specific chemicals suited for H_2_S removal can improve the adsorption process. Unfortunately, at a certain point, the adsorbents’ performance can be degraded after prolonged use in multiple cycles of the adsorption–desorption process, and they become no longer usable. Moreover, the performance of H_2_S adsorption capacity may drop up to 23% when other gases, such as CO_2_, are introduced into the same adsorber system [29]. 

Commonly, AC is difficult to reuse because the oxidation of sulfur polymers that are resistant to oxidation makes it nearly waterproof [40]. Replacing spent AC with new CAC is a typical practice in the industry to lower operating costs and address regeneration difficulties. Alternative strategies transform saturated adsorbents into fertilizers or value-added commodities, such as gypsum, to reduce destruction. In any case, further study is needed on the stability of the sulfur component after adsorption before the safe disposal of saturated adsorbents can be carried out. The use of agricultural waste as a H_2_S gas adsorbent is the first step towards an eco-friendly method, but the main challenge will be maintaining the finished product after removal.

Thus, the spent AC can be recycled through thermal processing or cold/hot water washing. A study on AC regeneration by cold and hot water purification at 300 °C in ambient air reported that H_2_S adsorption capacity fell by 60% due to adsorption on non-reverse chemicals in the active area [41]. In another study [40], the AC used as adsorbent for H_2_S adsorption was regenerated using nitrogen and heating for 10 min at 500 °C. A performance drop in AC can be avoided using this method, which can only be applied on a laboratory scale and could also be opened to industrial sizes (eliminating the opportunity for AC self-ignition). 

Therefore, in this study, metal-based adsorbents with varying active phase loadings are synthesized by modifying a commercial AC surface with a zinc acetate and zinc oxide reagent. Then, the adsorption–desorption cycles are implemented to ascertain the H_2_S captured to capture hydrogen sulfide (H_2_S) in order to examine the profile of adsorption–desorption and evaluate the H_2_S adsorption capacity. Moreover, all prepared adsorbents are characterized by nitrogen adsorption, Brunauer–Emmett–Teller surface area analysis, scanning electron microscopy–energy-dispersive X-ray spectroscopy, and Fourier transform infrared spectroscopy before and after the adsorption–desorption cycles. Furthermore, the behavioral model for H_2_S adsorption–desorption is proposed and validated using various analyses throughout adsorption–desorption samples. The relationship between the adsorbent’s characterization and behavioral model is discussed on the basis of the adsorption–desorption profile and H_2_S adsorption capacity.

## 2. Methodology

### 2.1. Materials

Commercial coconut shell activated carbon (CAC) with a granular shape was used as the main porous adsorbent in this study. Fresh AC samples were first sieved to 3–5 mm before modification by dual chemical mixture (DCM) and core–shell (CS) methods. Both methods require certain reagents, such as zinc acetate (ZnC_4_H_6_O_4_) at 98% purity, 99.5% zinc oxide (ZnO), 95% ammonia (NH_3_), and 98% tetraethyl orthosilicate (TEOS), all of which were purchased from Friendemann Schmidt Chemicals, Malaysia and used as-is without purification. Laboratory-scale experiments were conducted in a continuous atmospheric packed bed reactor or adsorber column with 0.5 cm diameter, 10 cm bed height, and 0.2 L total bed volume. Linde Malaysia Sdn Bhd provided 5000 ppm H_2_S-balanced N_2_ for this study’s feed gas. This gas concentration was selected based on the general H_2_S produced in the biogas system of palm oil mills. However, the exact biogas composition, including other gases (CH_4_, CO_2_, etc.), were not considered in this study as we focused on adsorbents’ development and their capability of capturing H_2_S gas. 

### 2.2. Adsorbent Modifications

AC modifications were conducted by DCM and CS methods as mentioned in detail by Zulkefli et al. [30] and Zulkefli et al. [31]. The basic preparations for the DCM and CS methods require CAC to be immersed in the designated solution prepared at 0.2 M concentration and continuously stirred for about 49 min up at 95 °C. The solution was then filtered, washed multiple times, and dried at 120 °C for 24 h through an oven unit. The DCM and CS adsorbents are denoted as ZnAc_2_/ZnO/CAC_DCM and ZnAc_2_/ZnO/CAC_CS, respectively, throughout this study.

### 2.3. H_2_S Adsorption–Desorption

Adsorption tests were conducted with constant feed concentration (5000 ppm H_2_S diluted in N_2_), flow rate (5.5 L/min), absolute pressure (1.5 bar), and temperature 30 °C (room temperature). Before H_2_S gas was introduced, 100 g of the prepared adsorbent was loaded into the column. For environmental safety, the outlet H_2_S concentration was maintained at 5 ppm. All output concentrations were measured by portable H_2_S analyzers (model GC310). Three adsorption–desorption cycles were performed on the adsorbents. For each desorption process, the adsorbent was regenerated at 150 °C. The details of the desorption process were previously described by Zulkefli et al. [29,30,31,32]. The general equations used for adsorption capacity and adsorbent performance degradation were adapted from a previous study [29]. The H_2_S adsorption capacity and degradation of adsorption in the next cycles were calculated based on an equation used as in a previous study [29,42].

### 2.4. Behavior Mechanism on Prepared Adsorbents and Adsorption–Desorption Process

This process was utilized to identify chemical, carbon, and H_2_S gas interactions that may influence H_2_S gas adsorption. Two main procedures were involved in the mechanism study of the selected adsorbents. The first mechanism was based on the pH changes that occur at each synthesis level. The pH conditions of distilled water, chemical solution, and CAC solution after 49 min were measured by a pH meter. All pH change data were recorded, and the characterization process was performed solely on the adsorbent that had been completed, and the adsorbents were labeled as DCM/CAC (F) and CS/CAC (F) for fresh adsorbents. 

Next, the behavior mechanism was determined through adsorption–desorption up to three cycles. The best adsorbents in this investigation were ZnAc_2_/ZnO/CAC_DCM and ZnAc_2_/ZnO/CAC_OS, which were analyzed using characterization techniques such as Brunauer–Emmett–Teller (BET) surface area analysis, scanning electron microscopy (SEM)–energy-dispersive X-ray spectroscopy (EDX), and Fourier transform infrared (FTIR) analysis in each cycle. The sampling process was observed on fresh adsorbents (F), the adsorbent saturated in the first cycle of adsorption (T1), the adsorbent desorbed in the first cycle (D1), the adsorbent saturated in the second cycle (T2), the adsorbent desorbed in the second cycle (D2), the adsorbents saturated in the third cycle (T3), and the adsorbents desorbed in the third cycle (D3). This method was only applied to the two most effective types of adsorbents. The mechanism study depended on the analysis of the adsorption–desorption process.

### 2.5. Behavior Mechanism Study

All of the synthesized adsorbents were subjected to numerous physical and chemical testing, including SEM, BET, FTIR, and thermogravimetric (TG) analyses. The surface morphology and chemical content of DCM adsorbent particles were observed with a scanning electron microscope (model EDAX APOLLO X, Mahwah, NJ, USA) and a CARL ZEISS EVO MA10 (Jena, Germany), respectively. The examination was conducted under a 10 kV accelerating voltage to reveal the details of adsorbent characteristics in terms of structure particles and the weight percentages of elements present on the adsorbents’ surfaces. Micrometric ASAP 2010 Version 4.0 was utilized in BET calculation (Micrometric, Lincoln, UK). The physical properties of the adsorbents were evaluated by N_2_ adsorption–desorption at 196 °C using a Quantachrome Autosorb 1 °C after 4 h of degassing at 150 °C. The precise surface was derived using BET calculation. The surface area was measured using the BET isotherm, and the pore volumes and standard pore volumes were computed using the N_2_ adsorption isotherm at P/P_0_ = 0.98. Prior to examination, powdered AC samples were combined with KBr. A PerkinElmer FTIR spectrometer was utilized to monitor FTIR spectra with a frequency range of 600–4000 cm^−1^.

## 3. Results and Discussion

### 3.1. Effect of pH and Their Behavior in Adsorbents Synthesize

The development of the selected adsorbents was also investigated to determine the effect of pH on the CAC modification process. In this section, two types of adsorbents were chosen: ZnAc_2_/ZnO/CAC_DCM and ZnAc_2_/ZnO/CAC_OS. pH variations throughout the saturation process were used to study the adsorbents. The adsorbents were produced in 0.22 M impregnation solution for 49 min at 35, 65 and 95 °C. 

The pH data are presented in Table 1. The pH became acidic at pH 4.23–5.6 after ZnAc_2_ was mixed with distilled water because the molecules come from acidic groups. Then, the pH decreased when ZnO compounds were mixed into the solution. This drop indicates that the acidity of the solution increased. The pH of the ZnAc_2_ and ZnO combination solution was likewise raised by the addition of raw CAC. pH levels considerably dropped after soaking for 49 min. This drop is a result of ZnAc_2_ and ZnO materials depositing on the CAC’s surface and pores. Furthermore, increasing the temperature during adsorbent synthesis also influenced the decrease in pH.

**Figure 1:**
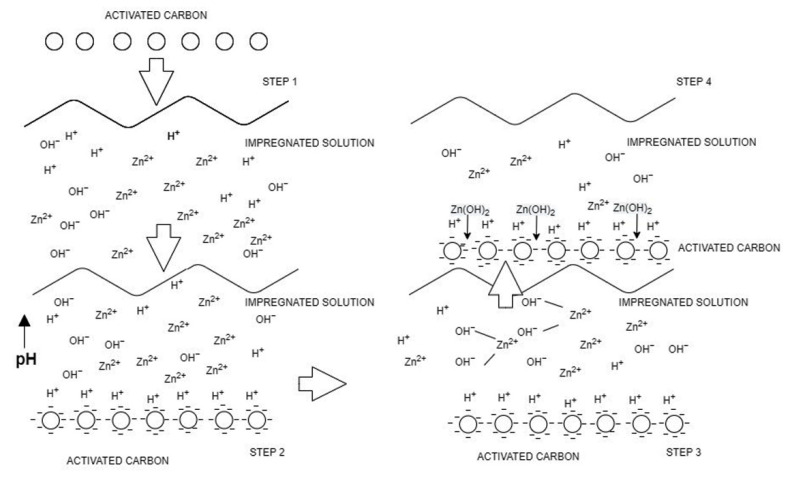
Impregnation step behavior of ZnAc_2_/ZnO/CAC_DCM.

Figure 1 depicts the ZnAc_2_ and ZnO modification processes that occur on the raw CAC surface. This adsorbent’s synthesis describes the likelihood of the mechanism involved during the saturation process. ZnAc_2_ and ZnO react to form Zn^2+^, O^2−^, OH^−^, and H^+^ ions. When the raw CAC was soaked in a compound solution, its strong negative charge attracted positive ions, particularly H^+^, resulting in an increase in pH value as indicated in Table 1. Zn^2+^ ions combine with hydroxyl to generate Zn(OH)_2_, which is then adsorbed on the CAC’s surface and pores.

**Figure 2:**
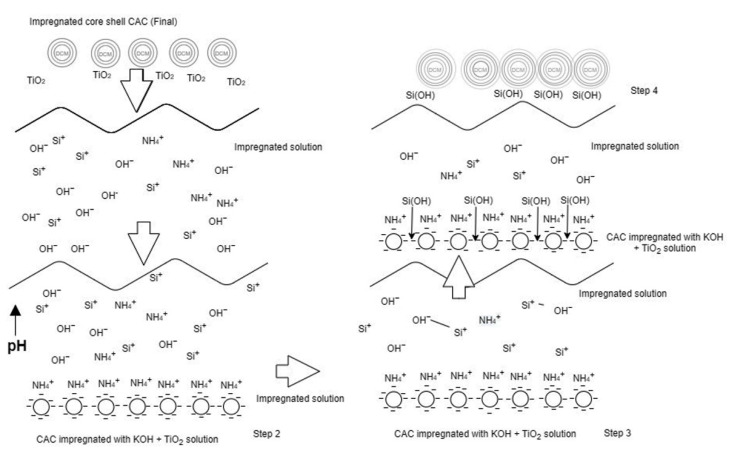
Impregnation step behavior of ZnAc_2_/ZnO/CAC_OS.

ZnAc_2_/ZnO/CAC_OS was prepared under the same parameter as ZnAc_2_/ZnO/CAC_DCM. The influence of pH on ZnAc_2_/ZnO/CAC_OS is shown in Table 2 and Figure 2. The pattern in the table reveals that preparation temperature had a major impact on the development of this adsorbent. The pH increased when CAC was added to a compound solution with other adsorbents. A remarkable drop in pH was also observed after 49 min of immersion, which was probably caused by chemicals being deposited on the CAC’s surface and pores.

The solution’s pH value decreased as the preparation temperature rose. This outcome may be due to the chemical breakdown of the molecule utilized. However, the production of additional adsorbents and the immersion of CAC into the solution increased the pH level. After 49 min of immersion, the pH readings started to fall, possibly as a result of chemicals being deposited on CAC surfaces and pores. 

### 3.2. Adsorption–Desorption Behavior

The mechanisms of the adsorption processes of ZnAc_2_/ZnO/CAC_DCM and ZnAc_2_/ZnO/CAC_OS were investigated in this work using multiple characterization techniques, such as SEM-EDX, BET, and FTIR. Characterization was performed three times for each grunting procedure. Each sample was labelled as described in Section 2.4.

(a) SEM-EDX analysis

**Figure 3:**
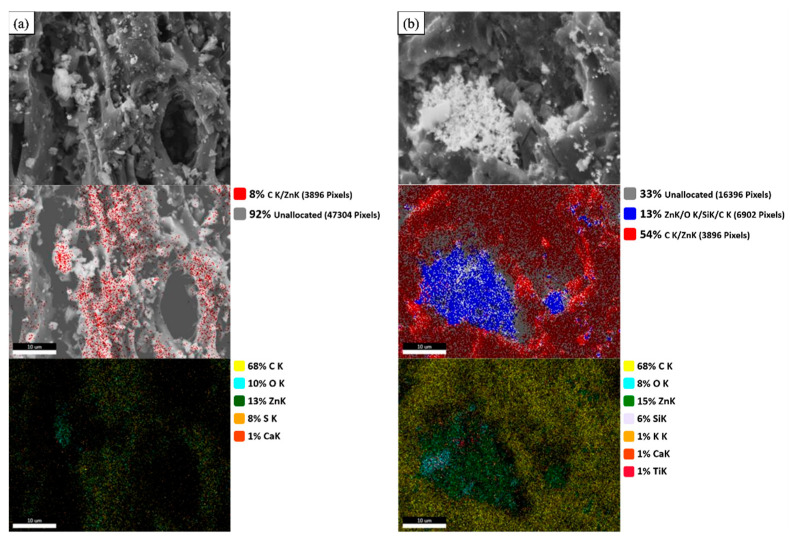
SEM mapping images of (**a**) ZnAc_2_/ZnO/CAC_DCM (F) and (**b**) ZnAc_2_/ZnO/CAC_OS (F) at 2.5K× magnification.

The morphological structures of fresh ZnAc_2_/ZnO/CAC_DCM and ZnAc_2_/ZnO/CAC_OS and the presence of elements on the adsorbents’ surfaces were performed using micrographic pictures and SEM mapping, respectively. Figure 3 depicts the morphological structure of each adsorbent below the 2.5K× magnification range and the 2-micron scale. The existence of colorful spots that form layers as a basis for the presence of chemical compounds on the surface of the adsorbent may be noticed in the ensuing image of the morphological structure observation. In general, the red spot on Figure 3a refers to the deposited overall elements including Zn and C, while Figure 3b shows the deposits of Zn, O, Si and C as presented by the blue spot. Additionally, the map shows the overall elements that were observed in detail, as presented by several other color spots. 

The deposition of coating layers on the surface of the adsorbent is believed to enhance gas adsorption. Thus, adsorbent addition via the saturation approach alters the morphological structure, which is believed to reduce the natural pores (based on the raw CAC) that are capable of enhancing the H_2_S-adsorbing surface area. In addition, observations of the adsorption–desorption cycle process were performed via data collected using EDX for the investigation of elements on the surfaces of DCM/CAC and CS/CAC for three complete cycles. Table 3 and Table 4 represent the elements that exist on the surface of the adsorbent depending on the mass percentage of the fresh adsorbent until the third adsorption cycle.

Among the elements found were carbon, calcium, zinc, oxygen, potassium, titanium, and sulfur. In accordance with the adsorbent obtained from carbon-type mesopores and the primary source of agricultural products, each adsorbent has carbon and calcium as components [43]. Carbon is the primary medium for this adsorbent; therefore, the amounts of carbon present on the surfaces of both adsorbents were considerably larger than the amounts of other elements. 

For the first cycle, the saturated adsorbent materials had a drop in zinc content (18.8% for ZnAc_2_/ZnO/CAC DCM and 25.9% for ZnAc_2_/ZnO/CAC OS). However, the total amounts of zinc elements required for breakdown dropped by 26% (ZnAc_2_/ZnO/CAC DCM) and 14.5% (ZnAc_2_/ZnO/CAC OS) in the third cycle. This drop is the result of the formation of ZnS on the surface of the adsorbent, which causes the decomposition of the decomposition cycle in preparation for the subsequent cycle.

The presence of moisture and oxygen has a substantial impact on effective adsorption. The reduction in the amount of oxygen element from fresh ZnAc_2_/ZnO/CAC_DCM to the saturated adsorbent in the first cycle was 28.6%, and the overall reduction in the amount of oxygen element in the third adsorption cycle was around 27%. The loss of oxygen during the first cycle in the saturated ZnAc_2_/ZnO/CAC_OS was 27%, whereas the loss of oxygen element during the entire process was only 15%. Compared with ZnAc_2_/ZnO/CAC_DCM, ZnAc_2_/ZnO/CAC_OS exhibited a lower oxygen element reduction, which is an indication that ZnAc_2_/ZnO/CAC_OS can be utilized more than once.

The presence of potassium and titanium elements was only detectable in ZnAc_2_/ZnO/CAC_OS when KOH and TiO compounds were used to synthesize the adsorbents During adsorption, K_2_S and TiS were formed, resulting in a decrease in saturation level. The drop was 16% for potassium element and 42% for element titanium element in the adsorption phase. This outcome is likely due to the fact that the TiO_2_ layer is the outermost layer following the TEOS layer, which is strongly bonded to the sulfur material. This result indicates that other layers may be reused multiple times and adsorb H_2_S gas over time. In addition, the fact that the silicon element on the outer layer decreased by 59% during the third adsorption cycle suggests that hazardous H_2_S gas is likely to damage the silica layer.

One may see an increase in ZnS production with each desorption step with increasing adsorption–desorption cycles. However, the ZnS increase in the ZnAc_2_/ZnO/CAC_DCM was more than that in ZnAc_2_/ZnO/CAC_OS (4.04% and 1.38%, respectively). The increase in ZnS on the surface of ZnAc_2_/ZnO/CAC_DCM may indicate a decline in performance over the subsequent cycle. Even after the adsorption process was completed, the sulfur substance, which affects the surface of the adsorbent, was difficult to remove. The presence of sulfur following the adsorption process was likewise consistent with the results of a previous study [43].

(b) BET analysis

**Figure 4:**
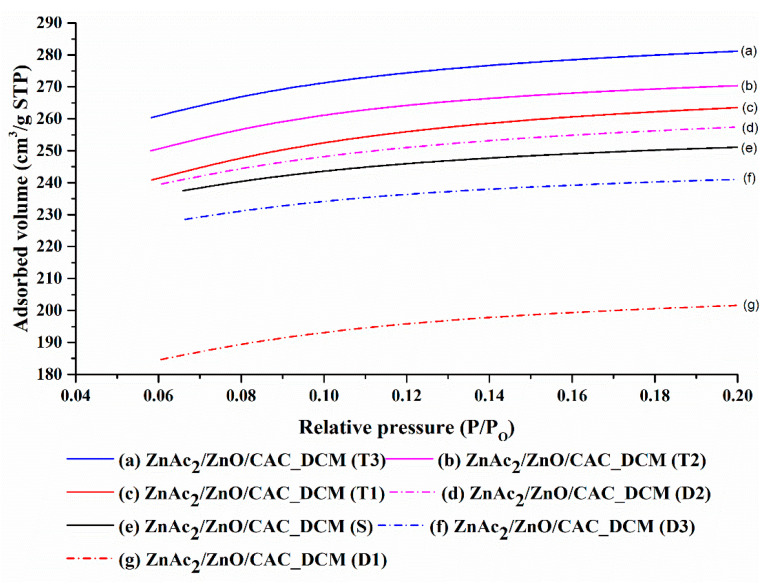
N_2_ adsorption–desorption isotherms of ZnAc_2_/ZnO/CAC_DCM in three H_2_S adsorption–desorption cycles.

Figure 4 depicts the adsorption of N_2_ gas as a function of pore volume in ZnAc_2_/ZnO/CAC_DCM. According to the IUPAC classification, the isothermal adsorption of N_2_ gas exhibits type I(b) isotherm with type IV hysteria, indicating that the adsorbent is formed by micropores [15,44,45]. Fresh adsorbents have a lesser volume of N_2_ adsorption than saturated and diabetic adsorbents. The adsorbent becomes saturated and increases in surface area, pore volume, and pore size during H_2_S gas adsorption, because the micropore area is blocked with metal compounds and H_2_S [37,46].

The desorbed adsorbents (D1, D2, and D3) showed a decrease in the volume of N_2_ adsorption compared with the saturated adsorbents (T1, T2, and T3). The procedure of dissolving at 150 °C and the combination of air regeneration agents and N_2_ demonstrated a positive influence on the next cycle’s adsorption efficiency. This decrease correlates with the findings of Shi et al. [47], who observed a decrease in adsorbent (AC) decomposition throughout the N_2_ adsorption study. In addition, pore structure was detected via the computation of isothermal N_2_ adsorption, and average pore volume was estimated using N_2_ adsorption at the relative pressure of 0.98. Micropore volume was also determined using the t-plot method. Table 5 provides a summary of the comprehensive pore structure, including the BET surface area (*S*_BET_), average pore volume, micropore area, and pore size.

A study of the BET surface areas and adsorbent pore counts for each of the adsorption–desorption processes revealed a decrease in comparison with ZnAc_2_/ZnO/CAC_DCM (F), with the exception of D1 and D3 adsorbents. The considerable drop in N_2_ adsorption volume was probably caused by metal particles obstructing the surface of the pores in the deepest pores [48]. Various essential issues must be addressed, such as the assessment of adsorption material regeneration, to keep the effectiveness of H_2_S gas adsorption for a period of the adsorption-desorption cycle. This finding is a result of the vapor’s probable performance degradation with each grunt cycle. The loss of surface area and obstruction of active sites on the surface of adsorbents because of multiple vapor–pore cycles may lead to a potential reduction in the efficiency of H_2_S gas adsorption.

The micropore area of the T1 adsorbent increased compared with the ZnAc_2_/ZnO/CAC_DCM (F) adsorbent owing to the presence of H_2_S particles within the pores of the adsorbent. The rise in micropore area and pore volume is indicative of H_2_S gas adsorption. The adsorbent in T1 phase was then degraded, and a decrease in surface characteristics was observed in the D1 adsorbent. The reduction in micropore area was 26% compared with that of fresh raw CAC. This result indicates that the adsorbents of type ZnAc_2_/ZnO/CAC_DCM may sustain 74% adsorption capability in the subsequent cycle. Compared with ZnAc_2_/ZnO/CAC_DCM, the adsorbent’s performance degraded through the micropore area at 1.2% and 3.1% during the second and third adsorption cycles, respectively.

The presence of metal in an adsorbent has no effect on the pore size factor. However, in this study, things are different. H_2_S gas adsorption is impacted by variations in pore size. In comparison with fresh and dehydrated adsorbent, the saturated adsorbents (T1, T2 and T3) had smaller pores. This finding demonstrates how well this adsorbent works. The results of the adsorption–desorption cycle, which are deteriorated for each cycle, can be matched by the low adsorption capability of this low pore size.

**Figure 5:**
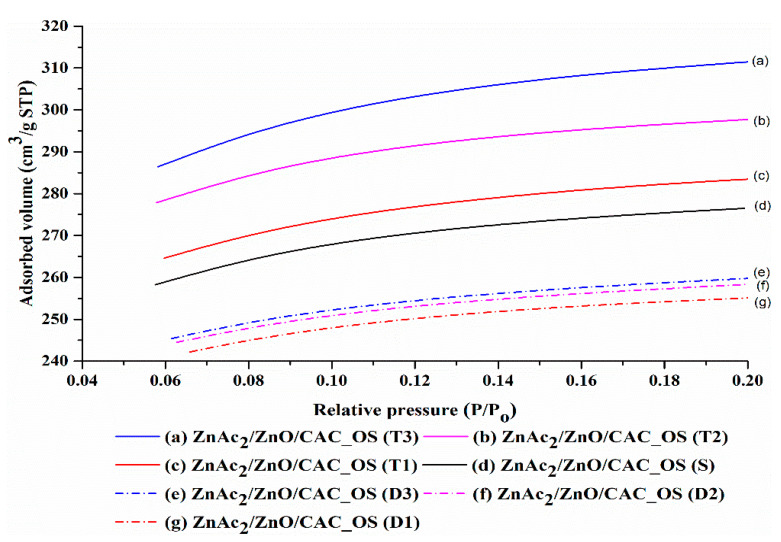
N_2_ adsorption–desorption isotherms of ZnAc_2_/ZnO/CAC_OS adsorbents through three H_2_S adsorption–desorption cycles.

Furthermore, surface characterization and N_2_ gas adsorption investigations were performed on ZnAc_2_/ZnO/CAC_OS materials to investigate its adsorption–desorption processes. Its N_2_ isotherm adsorption based on IUPAC was identical to the type 1 investigation. ZnAc_2_/ZnO/CAC_OS (F) was compared with its saturated (T1, T2 and T3) and desorbed counterparts (D1, D2, D3). All saturated materials (T1, T2 and T3) exhibited higher N_2_ adsorption than the fresh adsorbent as displayed in Figure 5. The result indicates that the H_2_S gas adsorbent on the ZnAc_2_/ZnO/CAC_OS surface has a higher surface area, micropore area, and pore volume for adsorption [37,46]. 

The N_2_ adsorption–desorption isotherm has acted upon the pore structure characteristics of ZnAc_2_/ZnO/CAC_OS that experienced three adsorption–desorption cycles. Each adsorption-desorption procedure of ZnAc_2_/ZnO/CAC_OS was calculated using BET analysis. Nitrogen intake at P/P_0_ = 0.98 was used to calculate the pore volume. The volume of micropores was determined using the t-plot approach. Table 6 provides a summary of the detailed pore structure of the parameter, including *S*_BET_, micropores volume, and pore size.

*S*_BET_ increased after H_2_S gas adsorption. The increase in *S*_BET_ for ZnAc_2_/ZnO/CAC_OS (T1) was 13% more than that for ZnAc_2_/ZnO/CAC_OS (T2) compared to ZnAc_2_/ZnO/CAC_OS (F). Among the exhausted adsorbents, ZnAc_2_/ZnO/CAC_OS (T1) exhibits a 19.1% decrease in *S*_BET_ compared with ZnAc_2_/ZnO/CAC_OS (D1). This phenomenon can be observed in the next adsorption–desorption cycle. Furthermore, the ZnAc_2_/ZnO/CAC_OS (D1) had an 8.6% reduction in active site, which decreased the *S*_BET_ values compared with ZnAc_2_/ZnO/CAC_OS (F) and decreased the pore size by 0.21%. Compared with ZnAc_2_/ZnO/CAC_OS (F), ZnAc_2_/ZnO/CAC_OS (D2) and ZnAc_2_/ZnO/CAC_OS (D3) were diminished by 5.1–5.7%. Hence, the reduction in ZnAc_2_/ZnO/CAC_OS in three adsorption–desorption cycles was stable and not excessive.

ZnAc_2_/ZnO/CAC_OS, in particular, is able to maintain a micropore area of 93.7% following a regeneration procedure compared with the micropore of fresh adsorbents, and its reduction in the next 2–3 cycles was negligible. This micropore area was substantial in the H_2_S gas adsorption process because it contains active sites that function effectively for effective H_2_S adsorption. Furthermore, the amount adsorbed in ZnAc_2_/ZnO/CAC_OS pores contributed to the reduction in surface area. Thus, chemical interactions with a reduction in micropore area and physical adsorption influence the H_2_S removal capability of the ZnAc_2_/ZnO/CAC_OS surface, which resulted in a decrease in pore size. This result is consistent with the findings of Wang et al. [49], Shi et al. [47], and De Oliveira et al. [15], who discovered that the regeneration of this adsorbent is dependent on the micropore area.

(c) FTIR analysis

After the adsorption–desorption process, a reduction in pore volume makes the chemical adsorption process for H_2_S hazardous to the adsorbent [15,50]. Chemical adsorption is influenced by a functional group of hydrophilic carbon, surface pH, and the presence of moisture film on the surface of the adsorbent; therefore, the reduction in pore volume can be demonstrated by a number of other characterization methods [15,49,50]. 

**Figure 6:**
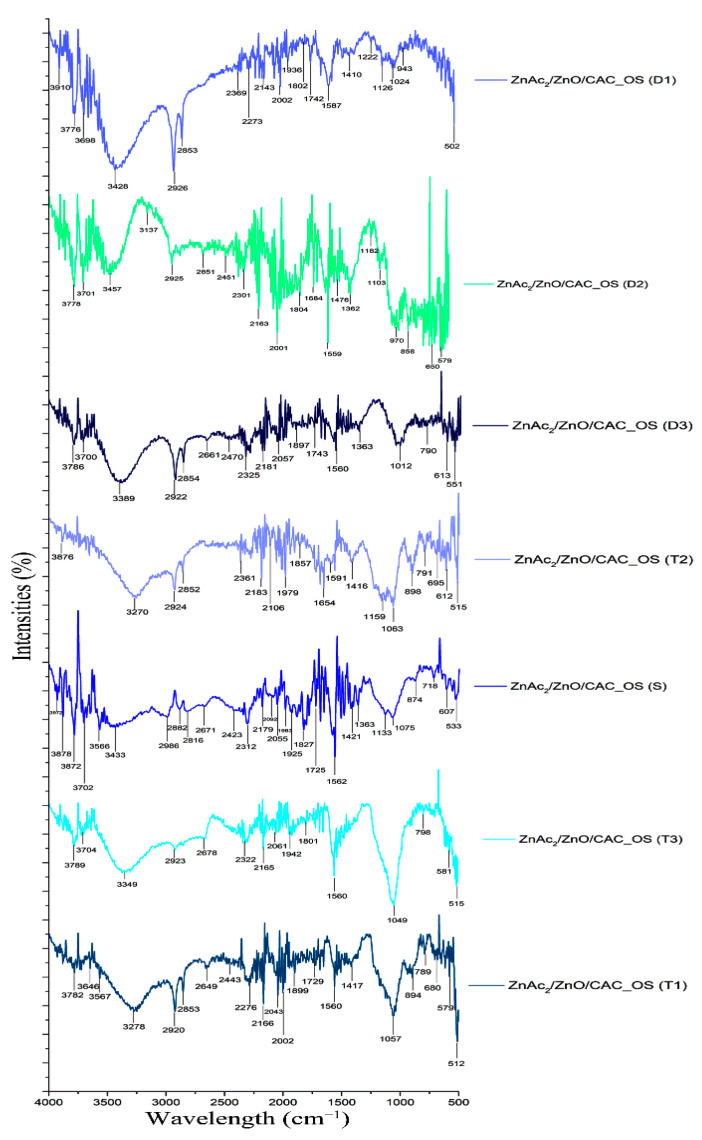
FTIR spectra of ZnAc_2_/ZnO/CAC_DCM through three H_2_S adsorption–desorption cycles.

**Figure 7:**
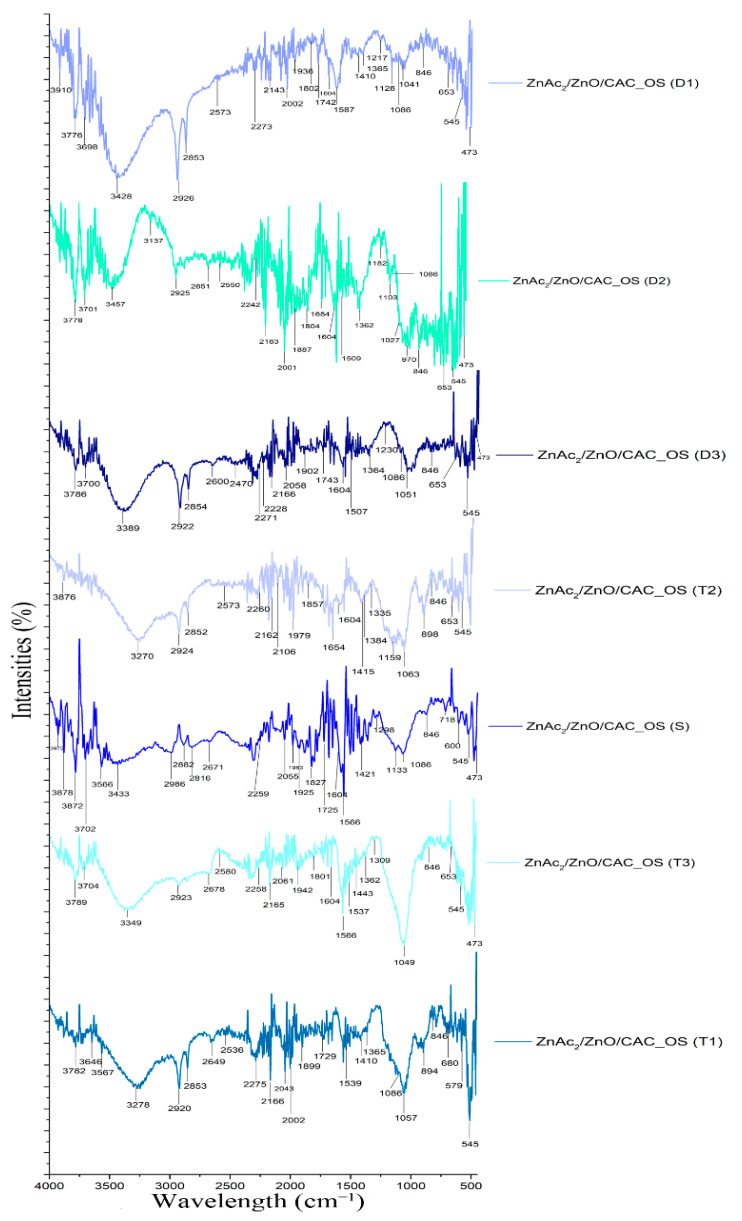
FTIR spectra of ZnAc_2_/ZnO/CAC_OS through three H_2_S adsorption–desorption cycles.

This chemical adsorption can be demonstrated by identifying the functional groups on the surface of the adsorbent using FTIR analysis. The comparison of these functional group pathways is attributable to the H_2_S gas adsorption mechanism of two adsorbents: ZnAc_2_/ZnO/CAC_DCM (Figure 6) and ZnAc_2_/ZnO/CAC_OS (Figure 7). Samples were collected at each adsorption (T1, T2, T3) and desorption step (D1, D2 and D3) to identify the functional groups on the surface of the adsorbent.

Few repeating peaks were identified and previously addressed. These peaks correspond to the hydroxyl group (OH), alkene (C–H), and carbonyl groups (C=O). Additionally, the chemical impregnated onto the adsorbent surface with metal oxide materials, such as ZnO, was also discovered at peaks between 1400 and 1600 cm^−1^ [51]. The presence of oxygen produced peak differences in the chemical adsorbents of ZnO, NH_3_, TEOS, KOH, and TiO_2_, showing that functional groups, such as carboxylic acid (O–H), ether (strain C–O–C), hydroxyl (O–H), acid anhydride (RC [=O] OC [=O] R), and the aromatic group (C=C ring) are at the peak wavelengths of 1550–1200 cm^−1^ [52,53]. 

The peak detection at 405 cm^−1^ for the Zn–C bond shows evidence of the interaction between the phase of metal material that was linked with the CAC [54]. The 470 cm^−1^ apex corresponds to Zn–O vibration [55]. Furthermore, the peaks at 418 cm^−1^ and 1400–1600 cm^−1^ are reserved for O–H and Zn–O, respectively [51]. The Zn–O path, however, vanished after the desorption process. After the adsorption procedure, the Zn–S functional group was found on the vibration band at 961 cm^−1^ [53,56]. However, the metal oxide route returns in the FTIR spectrum of the adsorbed substance. This finding demonstrates that the chemical link between the metal atom and atomic oxygen is broken during the desorption process, which is consistent with the earlier EDX research.

The carbon surface’s hydroxyl groups (OH) promote SO_2_ binding through ion–dipole interactions with COS. Therefore, the activity and selectivity of the concentrated compounds on precursors, such as AC, are remarkably impacted by the presence of functional groups in the oxidation reaction of H_2_S catalysts [57]. The Zn–S vibration band may be seen at 961 cm^−1^ [53,56]. The peak also supports the decryption of the EDX characterization of the sulfur element on the adsorbent, which states that the chemical link between the metal and oxygen atom is changed to the metal and sulfur atoms during the sulfidation process.

Adsorbents that have adsorbed gas under saturated circumstances (T1, T2 and T3) can be found in a variety of functional groups when compared with fresh adsorbents, particularly ZnAc_2_/ZnO/CAC_DCM (F). H_2_S gas adsorption exhibits numerous peaks involving sulfur bonds, including sulfate (S=O), sulfur oxide (S=O), and sulfonate (S=O), having peaks of 1415–1380, 1070–1030, and 1372–1335 cm^−1^, respectively. Peaks for weak aromatic groups with C–H swelling developed along the 2000–1650 cm^−1^ peak. The wavenumbers for saturated and diabetic adsorbents (D1, D2, and D3) were identical in terms of the functional groups present but differ in terms of wavenumber percentage of intensity. The percentage of intensity for saturated adsorbents was often higher than that for the adsorbed adsorbent.

Furthermore, additional functional groups for ZnAc_2_/ZnO/CAC_OS were added, such as N–H bonding of amine salt (3000–2800 cm^−1^), C–N bond strain weak for nitrile group (2260–2222 cm^−1^), N–H swelling band at 1650–1580 cm^−1^, the path of thiocyanate group with strong S–CN stretching (2175–2140 cm^−1^), and isotiocyanate (2140–1990 cm^−1^). The 1086 cm^−1^ strip depicts cyclic Si–O–Si bonding, whereas the 473 cm^−1^ strip depicts the SiO pathway [58]. The ZnAc_2_/ZnO/CAC_OS adsorbent had a lower intensity band than the ZnAc_2_/ZnO/CAC_DCM adsorbent, which has an amine functional group on the adsorbent. This amine group is thought to protect the Si–OH group from stretching vibrations and improve the adsorbent’s thermal stability on the adsorbent’s surface. It is associated with asymmetric bending and primary amine symmetry (–NH_2_) in the 1564 and 1480 cm^−1^ bands and secondary amine bending (–N[R]H) in the 1652 cm^−1^ band [59]. Finally, the functional group was analyzed in this adsorption process to illustrate the gas adsorption process via chemical adsorption, in which the groups interact with sulfur material on each saturated adsorbent and decompose.

### 3.3. Adsorbent Performance in H_2_S Adsorption–Desorption

**Figure 8:**
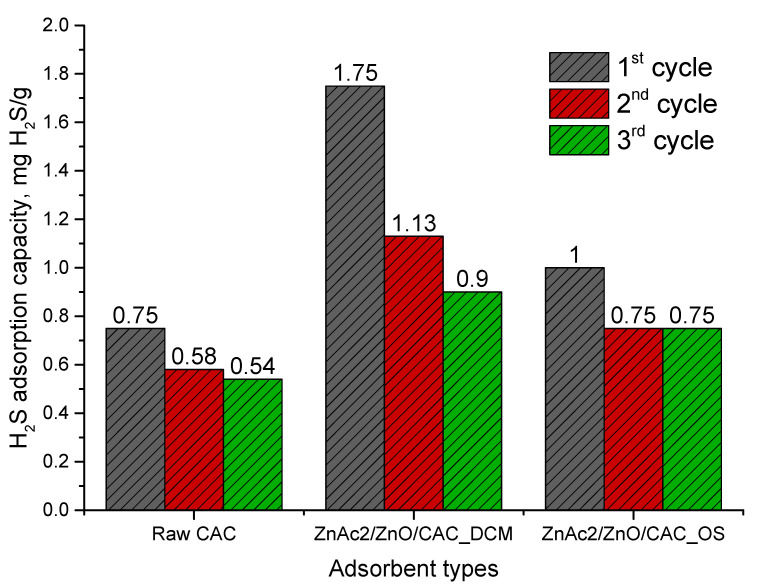
Adsorption–desorption profiles of raw CAC, ZnAc_2_/ZnO/CAC_DCM, and ZnAc_2_/ZnO/CAC_OS in three cycles.

The adsorption–desorption process was analyzed using ZnAc_2_/ZnO/CAC_DCM and ZnAc_2_/ZnO/CAC_OS to determine adsorption through the regeneration of saturated adsorption materials. Each adsorbent underwent desorption at 150 °C. The profile of ZnAc_2_/ZnO/CAC_DCM in three adsorption–desorption cycles is shown in Figure 8.

Each of these adsorbents performed well in the first adsorption–desorption cycle. This outcome is most likely due to the adsorbent’s surface pores having a high active site. In the first cycle, the amounts of H_2_S gas adsorption were approximately 1.75, 1.00, and 0.75 mg H_2_S/g for ZnAc_2_/ZnO/CAC_DCM, ZnAc_2_/ZnO/CAC_OS, and raw CAC, respectively. The second adsorption performance was observed after the first desorption process. The adsorption capacity in the second cycle decreased by 35.4% for ZnAc_2_/ZnO/CAC_DCM. While, 25% and 22.7% decrease were recorded in the second cycles of ZnAc_2_/ZnO/CAC_DCM. However, in the third cycle, the H_2_S gas adsorption of ZnAc_2_/ZnO/CAC_DCM and raw CAC kept decreasing by another 20.4% and 6.9%, respectively, whereas ZnAc_2_/ZnO/CAC_OS showed no changes in adsorption capability for current cycles. 

In conclusion, ZnAc_2_/ZnO/CAC_DCM had the capability to adsorb more H_2_S gas compared with other adsorbents; however, this adsorbent seems difficult to regenerate, as its adsorption capacity dropped drastically and was obviously not stable for longer adsorption–desorption cycles. However, ZnAc_2_/ZnO/CAC_OS performed better and was more stable compared with ZnAc_2_/ZnO/CAC_DCM and raw CAC. The decrease in adsorption capability with increasing adsorption–desorption cycles is caused by chemical interactions that reduce the adsorbent’s surface catalytic activity [59,60,61]. The interaction between sulfur and the adsorbent surface is one of factors that reduces H_2_S adsorption capability. Additionally, the production of stable sulfur polymers impairs adsorbent regeneration.

Ozekmekci et al. [60] suggested the application of a higher temperature in the desorption process to increase the adsorption capability in the next cycle as a good regeneration procedure that demands high temperatures of 500–600 °C in inert conditions. However, the researcher also advised to keep the adsorbent’s surface structure and effectiveness at temperatures below 400 °C [60]. Furthermore, based on the TG analysis of Zulkefli et al. [37,38,39,40], this temperature range might change the structure of the adsorbent’s surface, which could lessen its ability to adsorb H_2_S. 

**Figure 9:**
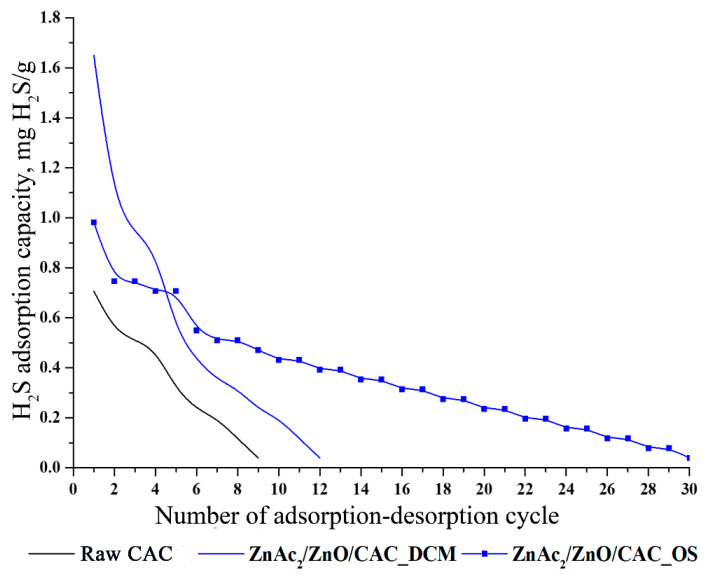
Maximum H_2_S adsorption–desorption profiles of ZnAc_2_/ZnO/CAC_DCM, ZnAc_2_/ZnO/CAC_OS, and raw CAC.

The successful adsorption–desorption of H_2_S in three cycles motivated us to observe further the capability of adsorbents until the maximum cycles. Raw CAC, ZnAc_2_/ZnO/CAC_DCM, and ZnAc_2_/ZnO/CAC_OS were evaluated for adsorption–desorption until they were saturated and could no longer be regenerated. Figure 9 depicts the comparison profile of the adsorbent up to the last adsorption–desorption cycle. 

According to Figure 9, the raw CAC was capable of regenerating up to the 9th cycle with a final adsorption capability of 0.04 mg H_2_S/g. In comparison, ZnAc_2_/ZnO/CAC_DCM and ZnAc_2_/ZnO/CAC_OS had outstanding adsorption capabilities with 12 and 30 cycles, respectively, and their final adsorption capacities were 0.04 mg H_2_S/g. Hence, ZnAc_2_/ZnO/CAC_OS showed the most prominent stabilization with a large number of cycles in the regeneration process. The stability of its adsorption–desorption cycles were 70% and 60% longer than those of raw CAC and ZnAc_2_/ZnO/CAC_DCM, respctively. As a result, ZnAc_2_/ZnO/CAC_OS had a higher number of regeneration cycles. Its adsorption capability can be further investigated to enhance its adsorption capacity in a longer regeneration cycle. 

## 4. Conclusions

Metal-based adsorbents with varying active phase loadings were synthesized to capture hydrogen sulfide (H_2_S). The behavior of adsorbents and the adsorption–desorption process were observed through pH changes and supported with several characterization analyses. The SEM-EDX, N_2_ adsorption–desorption isotherms, BET, and FTIR analysis were discussed based on physical changes toward selective adsorbents throughout three adsorption–desorption cycles. From the findings, modified adsorbents, dual chemical mixture (DCM) and a core–shell (CS) had the highest H_2_S adsorption performance with a range of 0.92–1.80 mg H_2_S/g. A further examination of the maximum adsorption–desorption cycle, i.e., more than 30 cycles, for each adsorbent type was performed until the minimum adsorption capacity of 0.04 mg H_2_S/g was recorded at the final adsorption cycles. The most stable and longer adsorption–desorption cycles with prominent capability of capturing H_2_S gas up to 70% more than raw CAC were ZnAc_2_/ZnO/CAC_OS. Even though the adsorption capacity of ZnAc_2_/ZnO/CAC_OS showed a 42.9% difference than ZnAc_2_/ZnO/CAC_DCM, further investigations can potentially be used to capture H_2_S gas with ZnAc_2_/ZnO/CAC_OS owing to their high efficiency, high stabilization, and economic feasibility. Meanwhile, the proposed behavioral model for H_2_S adsorption–desorption was validated effectively using various analyses throughout three cycles of adsorption–desorption samples. Furthermore, additional comprehensive analyses, i.e., X-ray Photoelectron Spectroscopy (XPS), and adsorption–desorption operation data, are required for the evaluation of the kinetics and mechanism of the H_2_S adsorption–desorption process.

## Figures and Tables

**Table 1:** pH value in each impregnation step of ZnAc_2_/ZnO/CAC_DCM at different soaking temperatures.

Impregnation Step	pH at 35 °C	pH at 65 °C	pH at 95 °C
Distilled water	7.00	7.00	7.00
ZnAc_2_ solution	5.60	4.92	4.23
ZnAc_2_ + ZnO solution	5.57	4.79	4.19
ZnAc_2_ + ZnO + raw CAC solution	5.74	5.04	4.82
Solution after 49 min	4.67	4.96	4.59

**Table 2:** pH value in each impregnation step of ZnAc_2_/ZnO/CAC_OS at different soaking temperatures.

Impregnation Step	pH at 35 °C	pH at 65 °C	pH at 95 °C
Distilled water	7.00	7.00	7.00
KOH solution	12.37	12.03	11.71
KOH + core (CAC) solution	12.89	12.63	12.35
Solution after 49 min	8.21	8.03	7.79
TiO_2_ solution	7.39	6.52	6.11
TiO_2_ + core (CAC) solution	7.61	7.06	6.74
Solution after 49 min	7.43	6.82	6.48
TEOS solution	6.39	6.58	5.73
TEOS + NH_3_ solution	9.13	8.81	8.70
TEOS + NH_3_+ core (CAC) solution	9.23	8.90	8.74
Solution after 49 min	7.69	7.34	7.29

**Table 3:** Element contents of the ZnAc_2_/ZnO/CAC_DCM surface in 3 cycles of H_2_S adsorption–desorption.

Adsorbent Samples	C	Ca	O	Zn	S
ZnAc_2_/ZnO/CAC_DCM (F)	38.07	0.17	26.75	35.01	0.00
ZnAc_2_/ZnO/CAC_DCM (T1)	30.93	1.22	19.11	28.43	20.31
ZnAc_2_/ZnO/CAC_DCM (D1)	39.34	0.79	25.77	33.27	0.83
ZnAc_2_/ZnO/CAC_DCM (T2)	29.67	1.73	20.14	27.19	21.27
ZnAc_2_/ZnO/CAC_DCM (D2)	40.11	0.80	25.21	32.67	1.21
ZnAc_2_/ZnO/CAC_DCM (T3)	28.42	1.90	18.75	28.54	22.39
ZnAc_2_/ZnO/CAC_DCM (D3)	49.61	0.88	19.56	25.91	4.04

**Table 4:** Element contents of the ZnAc_2_/ZnO/CAC_OS surface in three cycles of H_2_S adsorption–desorption.

Adsorbent	C	Ca	O	Zn	K	Ti	Si	S
ZnAc_2_/ZnO/CAC_OS (F)	27.46	0.67	37.27	23.73	1.88	1.59	7.40	0.00
ZnAc_2_/ZnO/CAC_OS (T1)	19.63	2.53	27.22	17.58	1.39	0.77	0.57	20.31
ZnAc_2_/ZnO/CAC_OS (D1)	36.87	0.81	33.02	21.67	1.80	1.47	3.57	0.79
ZnAc_2_/ZnO/CAC_OS (T2)	17.80	2.33	28.38	17.28	0.78	0.43	1.73	21.27
ZnAc_2_/ZnO/CAC_OS (D2)	38.41	0.94	32.19	21.35	1.60	1.36	3.28	0.87
ZnAc_2_/ZnO/CAC_OS (T3)	19.39	2.68	26.22	17.19	0.86	0.45	1.82	21.39
ZnAc_2_/ZnO/CAC_OS (D3)	40.03	0.91	31.84	20.29	1.58	0.93	3.04	1.38

**Table 5:** Porous properties of ZnAc_2_/ZnO/CAC_DCM in three H_2_S adsorption–desorption cycles.

Adsorbent Samples	*S*_BET_ (m^2^/g)	Average Pore Volume (cm^3^/g)	Micropore Area (m^2^/g)	Pore Size (Å)
ZnAc_2_/ZnO/CAC_DCM (F)	847.10	0.41	688.14	19.21
ZnAc_2_/ZnO/CAC_DCM (T1)	921.01	0.42	725.79	18.42
ZnAc_2_/ZnO/CAC_DCM (D1)	688.43	0.33	509.23	19.28
ZnAc_2_/ZnO/CAC_DCM (T2)	957.17	0.45	747.33	18.98
ZnAc_2_/ZnO/CAC_DCM (D2)	875.90	0.42	680.15	19.00
ZnAc_2_/ZnO/CAC_DCM (T3)	900.48	0.43	668.24	18.89
ZnAc_2_/ZnO/CAC_DCM (D3)	812.73	0.38	667.12	18.93

**Table 6:** Porous properties for ZnAc_2_/ZnO/CAC_OS through three H_2_S adsorption–desorption cycles.

Adsorbent Samples	*S*_BET_ (m^2^/g)	Average Pore Volume (cm^3^/g)	Micropore Area (m^2^/g)	Pore Size (Å)
ZnAc_2_/ZnO/CAC_OS (F)	940.41	0.45	756.53	19.17
ZnAc_2_/ZnO/CAC_OS (T_1_)	1062.23	0.51	805.52	19.11
ZnAc_2_/ZnO/CAC_OS (D_1_)	859.41	0.41	709.16	19.13
ZnAc_2_/ZnO/CAC_OS (T_2_)	1011.29	0.48	816.15	19.10
ZnAc_2_/ZnO/CAC_OS (D_2_)	877.91	0.42	717.59	19.25
ZnAc_2_/ZnO/CAC_OS (T_3_)	962.15	0.46	760.78	19.08
ZnAc_2_/ZnO/CAC_OS (D_3_)	872.16	0.42	713.69	19.10

## Data Availability

All relevant data are contained in the present manuscript.
